# Mapping Alzheimer's disease heterogeneity with molecular imaging biomarkers

**DOI:** 10.1111/eci.70163

**Published:** 2025-12-17

**Authors:** Elif Harput, Cecilia Boccalini, Gregory Mathoux, John O. Prior, Nathalie Testart, Mario Jreige, Valentina Garibotto

**Affiliations:** ^1^ Laboratory of Neuroimaging and Innovative Molecular Tracers (NIMTlab), Geneva University Neurocentre and Faculty of Medicine University of Geneva Geneva Switzerland; ^2^ Division of Nuclear Medicine and Molecular Imaging Geneva University Hospitals Geneva Switzerland; ^3^ Nuclear Medicine and Molecular Imaging Lausanne University Hospital Lausanne Switzerland; ^4^ CIBM, Centre for Biomedical Imaging University of Geneva Geneva Switzerland

**Keywords:** Alzheimer's disease, biomarkers, co‐pathologies, imaging, positron emission tomography

## Abstract

**Background:**

Alzheimer's disease (AD) is neuropathologically defined by the buildup of misfolded proteins such as extracellular amyloid‐β (Aβ) and intracellular tau neurofibrillary tangles. AD also extends beyond these pathological processes, and additional mechanisms such as synaptic dysfunction, microglial activity, astrocytic neuroinflammation play an important role as biomarkers of AD progression. In vivo evaluation and quantification of these molecular processes are possible with positron emission tomography (PET) imaging. As disease‐modifying therapies are entering clinical use, biomarkers' importance for early diagnosis and longitudinal monitoring of the disease increases.

**Results:**

Aβ is the earliest signature of AD which can be measured with PET imaging, followed by tau‐PET positivity, which is highly specific and central for staging and longitudinal monitoring. FDG‐PET continues to serve as a gold standard for detecting neurodegeneration, challenged by emerging dual‐phase PET protocols for amyloid and tau imaging, which integrate perfusion as a measure of neurodegeneration and pathology information in a single session, enhancing diagnostic efficiency. Synaptic density imaging reveals early synaptic loss linked to cognitive performance and decline. Neuroinflammation tracers can visualize microglial and astrocytic activation, contributing to disease onset and progression. Novel PET tracers targeting alpha‐synuclein and TDP‐43 show great promise for detecting co‐pathologies which can contribute to AD clinical heterogeneity.

**Conclusion:**

PET imaging has advanced the field by enabling visualization of AD‐related changes and providing measurable outcomes for clinical trials and disease‐modifying therapies. Imaging of related pathologies can further improve diagnostic accuracy and provide important insights into disease heterogeneity. Moving forward, integrating multiple PET biomarkers into personalized diagnostic approaches will be crucial.

## Graphical abstract

This review outlines positron emission tomography (PET) biomarkers used in Alzheimer's disease (AD) research and clinical settings, targeting both specific (amyloid, tau) and non‐specific (neurodegeneration, synaptic density, neuroinflammation) processes. Clinically validated amyloid and tau PET enable early diagnosis, prognosis and monitoring, while research tracers for neuroinflammation and synaptic density provide insights into disease heterogeneity. Molecular imaging of co‐pathologies such as alpha synuclein and TDP‐43 could improve diagnostic accuracy and novel molecules for these targets are under investigation. Integrating multiple biomarkers into personalized frameworks is essential for achieving better outcomes in drug development.

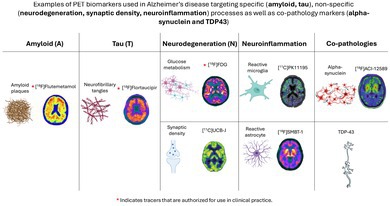

## 1 INTRODUCTION ON ALZHEIMER'S DISEASE AND POSITRON EMISSION TOMOGRAPHY

Alzheimer's disease (AD) is the leading cause of dementia and, from a neuropathological perspective, is characterized by the accumulation of abnormal proteins such as extracellular amyloid‐β (Aβ) forming senile plaques and intracellular hyperphosphorylated tau aggregating into neurofibrillary tangles (NFTs).^1^ Aβ accumulation may begin 20–30 years before clinical symptoms emerge, making early detection essential.^1^ According to the amyloid cascade hypothesis, Aβ accumulation initiates a series of neurobiological events culminating in tau aggregation, neurodegeneration and ultimately cognitive decline.^1^ However, AD is not limited to amyloid and tau; other pathological processes such as synaptic dysfunction, microglial activation and inflammation also contribute significantly to the disease pathology.^2^ Molecular imaging proposed by the National Institute on Aging – Alzheimer's Association (NIA‐AA), which emphasizes biomarker‐based definitions over clinical syndromes, plays a central role in the revised biological framework for AD.^3^

**FIGURE 1:**
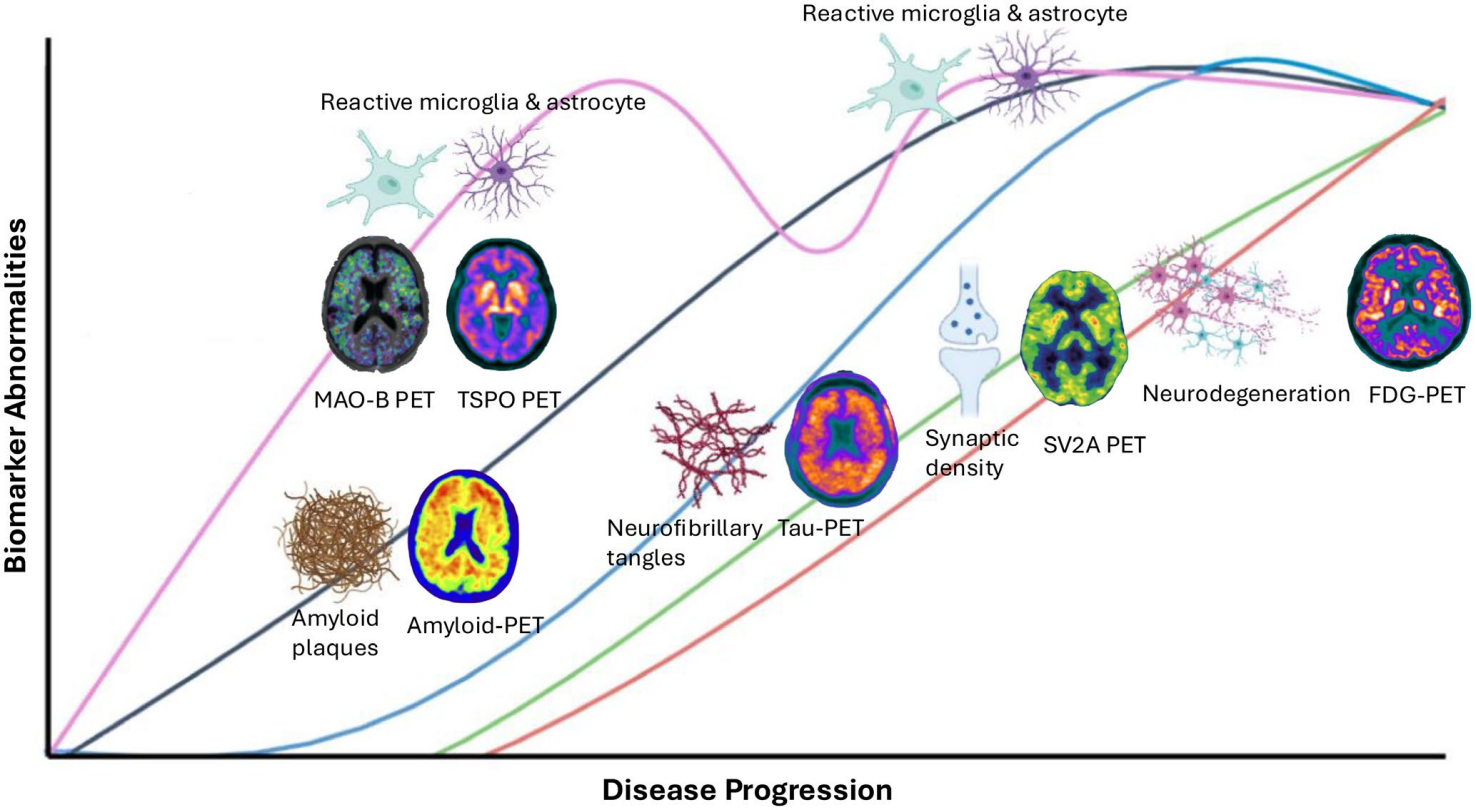
Temporal progression of biomarker abnormalities in AD, including both AD‐specific and non‐AD biomarkers and their corresponding PET imaging applications across the disease continuum.

The AT(N) classification system provides a biological framework for categorizing biomarkers into three core groups: A for amyloid pathology, T for tau pathology and N for neurodegeneration, while offering the flexibility to include emerging biomarkers.^2^, ^4^ Beyond the core biomarkers of AD, they can also be grouped into non‐specific biomarkers and markers of common non‐AD pathologies. These biomarkers can be assessed using different neuroimaging techniques, as illustrated in Figure 1. Molecular imaging with positron emission tomography (PET) serves for in vivo visualization and quantification of pathology, providing essential information on its location and distribution. With the emergence of new treatments, biomarkers play a crucial role in identifying appropriate patient populations and monitoring therapeutic outcomes. Moreover, molecular imaging is rapidly evolving in AD research with growing efforts to image novel targets with innovative radiotracers.^5^

The scope of this review is to present an overview of molecular imaging biomarkers in AD as summarized in Table 1. First, we discuss biomarkers that are specific to AD pathology, then we present non‐specific biomarkers for associated processes, and lastly, we introduce biomarkers for co‐pathologies.

**TABLE 1:** Summary of biomarkers, their pathological target, role in the AT(N) classification, tracers and their sensitivity/specificity values.

Biomarker	Pathological target	Role in the AT(N) classification framework	Tracer name	Sensitivity/specificity
Amyloid	Fibrillar Aβ plaques	Amyloid (A)	[^11^C]PiB [^18^F]Florbetapir^a^ [^18^F]Flutemetamol^a^ [^18^F]Florbetaben^a^	97%/73%^135^ 92%/91%^136^ 91%/88%^137^ 98%/89%^138^
Tau	Paired helical filament tau	Tau (T)	[^18^F]Flortaucipir^a^ [^18^F]MK6240 [^18^F]RO948 [^18^F]PI‐2620	93%–100%/52%–92%^139^ 79%/82%^140^ 91.9%/60.6%^141^ >85%/100%^142^
Neurodegeneration	Glucose metabolism Synaptic density	Neurodegeneration (N)	[^18^F]Fluorodeoxyglucose^a^ [^18^F]SynVesT‐1 [^11^C]UCB‐J [^11^C]UCB‐H	94%/81%^135^
Neuroinflammation	Microglia (TSPO) Astrocytes (MAO‐B)	Neuroinflammation (I)	[^11^C]PK11195 [^18^F]DPA‐714 [^11^C]PBR28 [^11^C]DPA‐713 [^18^F]FEDAA1106 [^18^F]FEPPA [^11^C]DED [^18^F]SMBT‐1	
Alpha‐synuclein	Lewy bodies	Co‐pathology	[^18^F]F0502B [^18^F]C05‐05	
TDP‐43	TDP‐43 inclusions	Co‐pathology	[^18^F]Fluorodeoxyglucose [^18^F]ACI‐19626	

*Note*: The sensitivity and specificity of each tracer (with the exception of [^18^F]MK‐6240, [^18^F]RO‐948 and [^18^F]PI‐2620) were calculated relative to post‐mortem confirmation, reflecting their performance in distinguishing Alzheimer's disease from healthy subjects.

Abbreviations: MAO‐B, Monoamineoxidase B; TDP‐43, TAR DNA binding protein 43; TSPO, Translocator protein.

^a^ Indicates tracers that are authorized for use in clinical practice.

## 2 SPECIFIC BIOMARKERS FOR AD PATHOLOGY

### 2.1 Amyloid (amyloid‐PET)

Aβ deposition in the brain marks the earliest critical event in the pathological progression of AD. Amyloid plaques represent the extracellular accumulation and deposition of Aβ, which is a product of the processing of the amyloid precursor protein.^6^ Deposition usually starts in the posterior cingulate, retrosplenial cortex and precuneus regions.^1^, ^7^ Thal phases of Aβ accumulation describe the sequential progression of amyloid‐β deposition in the brain, beginning in neocortical areas and extending to subcortical regions.^8^ The introduction of amyloid‐PET has dramatically enhanced the field of molecular neuroimaging by allowing direct and non‐invasive visualization of Aβ deposition in the living brain.

Amyloid‐PET imaging defines the ‘A’ component in the AT(N) classification system, and it enables in vivo detection of Aβ plaques that may precede dementia by decades. This modality has been validated against histopathological standards, and studies have confirmed its correlation with the classical neuropathological staging systems.

The first amyloid‐PET tracer to demonstrate its feasibility in humans was [^11^C]PiB (Pittsburgh Compound‐B), with high affinity for fibrillar Aβ plaques. Although [^11^C]PiB remains the gold standard in research, its clinical utility is limited because of the 20‐min half‐life of carbon‐11.^9^ This limitation has catalysed the development of fluorine‐18‐labelled radiotracers, including [^18^F]Florbetapir, [^18^F]Florbetaben and [^18^F]Flutemetamol, which are approved by the U.S. Food and Drug Administration (FDA) and European Medicines Agency (EMA) and are widely available for clinical use (https://www.ema.europa.eu/en/documents/product‐information/amyvid‐epar‐product‐information_en.pdf; https://www.ema.europa.eu/en/documents/product‐information/neuraceq‐epar‐product‐information_en.pdf; https://www.ema.europa.eu/en/documents/product‐information/vizamyl‐epar‐product‐information_en.pdf).^11^ Each tracer exhibits distinct pharmacokinetics, image contrast properties and degrees of non‐specific white matter binding, which necessitate careful selection based on institutional expertise and logistical factors. Table 2 provides a comparative overview.

**TABLE 2:** Summary of the main characteristics of FDA‐approved amyloid‐PET tracers used in clinical practice, including regulatory approval year, physical properties, injection and acquisition protocols and visual interpretation criteria based on established manufacturer and guideline recommendations.

Tracer name	FDA approval year	Half‐life	Typical injected dose	Acquisition start time	Scan duration	Interpretation criteria
[^18^F]Florbetapir^a^	2012	~110 min	~370 MBq (10 mCi)	50 min post‐injection	10–20 min	Cortical uptake > white matter in ≥2 regions
[^18^F]Flutemetamol^a^	2013	~110 min	~185 MBq (5 mCi)	90 min post‐injection	10–20 min	Increased uptake in ≥1 of: frontal, precuneus/posterior cingulate, lateral temporal, parietal, striatum
[^18^F]Florbetaben^a^	2014	~110 min	~300 MBq (8.1 mCi)	90 min post‐injection	15–20 min	Increased cortical uptake in ≥1 of: frontal, lateral temporal, parietal, posterior cingulate/precuneus; reference = cerebellum

Abbreviations: AD, Alzheimer's disease; FDA U.S., Food and Drug Administration; MBq, Megabecquerel; mCi, millicurie.

^a^ Indicates tracers authorized for use in clinical practice.

Head‐to‐head studies comparing [^18^F]Florbetaben and [^18^F]Flutemetamol tracers found that cortical amyloid uptake measured with the two tracers was highly correlated with each other across the brain, except for subcortical regions where [^18^F]Flutemetamol showed higher uptake in the striatum; however, when analyses were stratified by disease stage, the difference in amyloid uptake between tracers was found significant in the MCI stage. Moreover, the correlation between amyloid uptake measured with [^18^F]Flutemetamol and the rate of cognitive decline was stronger than that observed with [^18^F]Florbetaben.^10^, ^11^

Despite these differences, comparative studies have demonstrated a strong concordance among tracers in the binary classification of amyloid status. Nevertheless, subtle variations may influence the quantification thresholds and image interpretation criteria.^12^, ^13^ All three fluorinated tracers have established visual reading protocols which refer to a standardized binary assessment of the images by a trained observer providing a robust foundation for routine use.^14^

Although visual reads remain the clinical standard for amyloid‐PET, quantitative methods improve sensitivity, reproducibility and longitudinal tracking.^15^ Standardized uptake value ratio (SUVR) is the most common metric, comparing tracer uptake in cortical targets to a reference region (e.g. cerebellum), but its value depends on tracer kinetics, acquisition and analysis protocols, limiting comparability across sites.

To address these limitations, the Centiloid Project was launched to provide standardized measurements across tracers and centers. The scale, which ranges from 0 (young healthy controls) to 100 (typical uptake in AD patients), is validated for [^11^C]PiB and all major [^18^F] tracers, and it is widely adopted in multicentric studies and trials (e.g. ADNI, A4, EPAD). This harmonization enables consistent amyloid‐positivity thresholds and supports treatment monitoring.^16^ In a diagnostic setting, amyloid‐PET offers significant value in clarifying etiological uncertainty, particularly in cases with atypical presentations, early onset dementia, or mixed pathologies. In individuals with mild cognitive impairment (MCI) and cognitively unimpaired, amyloid‐PET positivity is associated with an elevated risk of progression to AD dementia.^17^, ^18^

Despite its strengths, amyloid‐PET may have limitations. Aβ burden correlates poorly with cognitive severity in later stages, where it has already reached a plateau.^19^ The majority of cognitively normal older adults are amyloid‐positive, which may complicate interpretation,^20^ and not all Aβ‐positive individuals progress to dementia within a decade,^21^ emphasizing the need for complementary markers. Finally, amyloid‐PET's high cost, limited access and reimbursement barriers restrict its widespread use, although health‐economic analyses suggest that it can reduce costs by preventing misdiagnosis and inappropriate treatment.^22^, ^23^

### 2.2 Tau (tau‐PET)

Tau‐PET has significantly enhanced our ability to assess tau pathology in vivo. In AD, tau aggregates form neurofibrillary tangles composed of paired helical filaments that follow a regional progression. Tau‐PET allows in vivo mapping of both the burden and anatomical distribution of tau pathology. In AD patients, tracer retention is markedly higher than in cognitively normal controls, most prominently in the inferior lateral temporal cortex, posterior cingulate and lateral parietal lobes, mirroring the regional patterns observed in histopathological studies.^24^

Braak and Braak proposed a sequential progression of tau pathology, starting from the medial temporal lobe towards widespread neocortical regions.^25^, ^26^ Building on this neuropathological staging, the NIA‐AA described a tau‐PET uptake‐based biological staging system that integrates amyloid status and the extent and intensity of tau‐PET signal, providing a standardized approach to stage AD in vivo.^27^

Different tracers have been developed in the last few years. First‐generation tracers include [^18^F]flortaucipir, [^11^C]PBB3 and the [^18^F]THK family.^28^, ^29^ Due to limitations such as off‐target binding, second‐generation tau‐PET tracers were introduced, such as [^18^F]MK6240, [^18^F]RO948, [^18^F]PI2620 and [^18^F]GTP1. They show greater affinity for neurofibrillary tau aggregates with less off‐target retention, enabling more reliable quantification of tau pathology.^30^ Currently, the only tau‐PET tracer approved by both the FDA and EMA for clinical use is [^18^F]Flortaucipir (https://www.ema.europa.eu/en/documents/product‐information/tauvid‐epar‐product‐information_en.pdf). Table 3 provides a comparative overview of the most used tracers, both in clinical and research settings.

**TABLE 3:** Summary of the main characteristics of tau‐PET tracers commonly used in clinical practice, including development status, physical properties, injection and acquisition protocols and visual interpretation criteria based on established manufacturer and guideline recommendations.

Tracer name	Developmental status	Half‐life	Typical dose	Uptake time	Scan duration	Off‐target binding	Visual interpretation criteria
[^18^F]Flortaucipir^a^	FDA and EMA approved (2020)	~110 min	~370 MBq (10 mCi)	80 min	20 min	Striatum, choroid plexus	Increase uptake in posterolateral temporal, parietal or occipital cortices, with or without involvement of the frontal lobes, consistent with Braak stages IV–VI.
[^18^F]MK6240	Investigational	~110 min	~370 MBq (10 mCi)	90–110 min	20 min	Meninges	Increase uptake in mesial temporal, inferior temporal and/or neocortical regions, classified as typical, limbic predominant, or MTL‐sparing patterns
[^18^F]RO948	Investigational	~110 min	~370 MBq (10 mCi)	60–90 min	20 min	Skull, meninges	Classified into four patterns: negative/non‐AD, temporal‐only (early AD), neocortical (late AD), or inconclusive; medial temporal uptake alone considered positive.
[^18^F]PI‐2620	Investigational	~110 min	~370 MBq (10 mCi)	45–75 min	20 min	Choroid plexus, venous and cavernous sinus	Ongoing development of AD‐specific diagnostic methods

Abbreviations: AD, Alzheimer's disease; EMA, European Medicines Agency; FDA U.S., Food and Drug Administration; MBq, Megabecquerel; mCi, millicurie; MTL, medial temporal lobe.

^a^ Indicates tracers authorized for use in clinical practice.

A few head‐to‐head comparative studies have been conducted. The comparison between [^18^F]flortaucipir and [^18^F]MK‐6240 suggested that both detect the same AD‐related tau pathology and align well in visual interpretation.^31^ Comparison of second‐generation tau‐PET tracers [^18^F]RO948 and [^18^F]PI‐2620 showed similar uptake patterns in all cortical regions. Both tracers were able to detect tau pathology in very early stages of the AD continuum.^31^, ^32^

However, due to the wide range of existing tracers, a unified and tracer‐agnostic quantitative framework is crucial to ensure valid and consistent comparisons across scans. Current initiatives, like CenTaur,^16^ are focused on developing and validating standardized scales, harmonizing measurements across different radiotracers and processing pipelines.^33^, ^34^ Such harmonization trials are essential for enabling tau‐PET in global diagnostic frameworks.

There are two different ways to interpret tracer uptake results: through visual interpretation or by semi‐quantitative measures. Quantitative analyses are mostly used in research settings; meanwhile, for clinical practice, visual interpretation is essential.^35^ Notably, studies have shown a strong association between visual and semi‐quantitative measures, with comparable diagnostic and prognostic performance.^36^

Although tau patterns strongly overlap with regions of atrophy, hypometabolism and correlate with cognitive performance, tau‐PET demonstrates high predictive accuracy for short‐term cognitive decline.^37^ Across studies, regional tau uptake explained a greater proportion of the variability in future MMSE decline than MRI‐derived atrophy or amyloid burden in amyloid‐positive cognitively unimpaired individuals.^37^ It also has outperformed amyloid‐PET, FDG‐PET and MRI in estimating cognitive and functional deterioration.^18^, ^30^, ^38^, ^39^, ^40^ The superior performance of tau‐PET may be particularly advantageous in symptomatic stages, where higher baseline tau predicts faster decline and helps distinguish variant AD subtypes characterized by distinct spatial patterns of accumulation.^13^, ^38^, ^41^ Tau‐PET has also revealed distinct spatiotemporal patterns associated with varying clinical presentations and differences in underlying neuroanatomy.^13^, ^42^, ^43^ Moreover, tau‐PET imaging holds significant promise as a biomarker to improve differential diagnosis and expand pathophysiological insights in non‐AD tauopathies, including frontotemporal lobar degeneration (FTLD), which includes Pick's disease, progressive supranuclear palsy (PSP) and corticobasal degeneration (CBD), with tracers like [^18^F]flortaucipir showing strong discriminatory power in differentiating AD and non‐AD diseases.^24^, ^44^ In cases with a negative tau‐PET scan, the relative pattern of brain atrophy may help identify the specific non‐AD aetiology.^45^ Moreover, tau‐PET positivity distinguishes AD‐related decline from cognitive impairment due to mixed or non‐AD pathologies, implying its value for differential prognosis.^46^

To conclude, tau‐PET is established across clinical and research settings, ensuring consistency in patient categorization, comparability with other biomarkers and ultimately supporting improved patient outcomes.

## 3 NON‐SPECIFIC BIOMARKERS FOR AD PATHOLOGY

### 3.1 Neurodegeneration

#### 3.1.1 FDG‐PET

Fluorodeoxyglucose positron emission tomography ([^18^F]FDG‐PET) is the most used neuroimaging tool measuring cerebral glucose metabolism and is considered the gold standard biomarker of neurodegeneration together with MRI. In 2024, NIA‐AA criteria for AD identified FDG‐PET and structural MRI as biomarkers reflecting non‐specific processes related to neurodegeneration in AD.^27^ FDG‐PET is a sensitive marker of neurodegeneration, synaptic and neuronal dysfunction, and it has demonstrated high accuracy in the early detection and staging of AD.^47^ Patterns of regional hypometabolism, observed early in the AD continuum, initially affect the posterior cingulate cortex and later spread to temporoparietal regions and frontal cortices.^47^, ^48^, ^49^ In many studies, semiquantitative analyses of FDG‐PET often focus on regions such as bilateral angular gyri, posterior cingulate and inferior temporal gyri, which are known to exhibit the characteristic hypometabolism observed in AD.^50^ Reductions in regional glucose metabolism in AD‐associated hypometabolic areas can be detected even before the appearance of structural brain changes in patients with AD.^49^

FDG‐PET demonstrates prognostic power in predicting clinical progression, particularly in identifying amyloid‐positive individuals who remain cognitively stable over time and in tracking conversion from MCI to AD dementia.^51^ Faster decline in glucose metabolism and lower baseline glucose metabolism were associated with more rapid cognitive decline and more pronounced metabolic differences between AD and MCI groups, compared to differences between MCI and cognitively normal, suggesting accelerating hypometabolism as AD progresses.^52^ Moreover, FDG‐PET utility extends across a spectrum of neurodegenerative diseases by capturing disease‐specific metabolic patterns that enhance diagnostic value and support its utility in distinguishing non‐AD dementias such as frontotemporal lobar degeneration or dementia with Lewy bodies.^53^, ^54^ According to the European Federation of Neurological Societies, in suspected cases of progressive supranuclear palsy and in cases of cortico‐basal syndrome, FDG‐PET is considered the primary biomarker. For frontotemporal lobar degeneration, a typical and distinctive pattern of hypometabolism is supported to confirm the diagnosis and complete the diagnostic evaluation.^55^ Compared to CSF biomarkers (CSF p‐tau181, t‐tau181, Aβ42/40), FDG‐PET demonstrates superior accuracy in differentiating AD from non‐AD disorders.^47^ When assessing the ability to predict progression to AD, amyloid‐PET has been reported to show slightly higher sensitivity compared to FDG‐PET, whereas FDG‐PET demonstrates greater specificity and superior accuracy to predict short‐term progression.^53^

Moreover, FDG‐PET is a valuable tool for differentiating atypical forms of AD characterized by specific hypometabolic patterns. In patients with the logopenic variant of primary progressive aphasia, hypometabolism primarily occurs in the posterior temporal cortex and inferior temporal lobule, with a predominance in the left hemisphere. In posterior cortical atrophy, hypometabolism involves the bilateral occipital and parietal cortices. Individuals with behavioural variants of AD show predominant temporoparietal hypometabolism that can extend to a mixed frontal and temporoparietal or predominant frontal pattern.^18^ These distinct patterns of hypometabolism in AD variants are closely linked to their clinical manifestations, facilitating earlier and more accurate diagnosis, which is often recognized later than in typical AD.^56^

#### 3.1.2 Dual phase protocol

Dual‐phase amyloid and tau‐PET imaging includes an early acquisition capturing tracer distribution immediately after injection as a perfusion measure and a standard late‐phase acquisition depicting protein accumulation. It has emerged as a promising approach in AD as it offers the possibility of having two pieces of information (neurodegeneration as perfusion and amyloid/tau pathological information depending on the tracer) with a single tracer injection.^57^ Thanks to the high lipophilicity of amyloid and tau tracers such as [^18^F]Florbetapir, [^18^F]Florbetaben, [^18^F]Flutemetamol, [^18^F]Flortaucipir, characterized by an elevated first‐pass influx rate (K1), early‐phase images depict cerebral perfusion, which is a measure closely linked to neuronal dysfunction through metabolic consumption.^57^, ^58^ Across multiple studies, early‐phase acquisitions were defined immediately post‐injection and lasted between 5 and 10 min.^59^, ^60^, ^61^ Early‐phase images of amyloid‐PET have shown strong visual and quantitative comparability with FDG‐PET, demonstrating similar regional uptake patterns.^58^, ^60^, ^61^, ^62^, ^63^ These perfusion patterns can serve as a surrogate marker for synaptic activity and metabolic function, thus providing valuable information about neuronal injury.^60^, ^61^, ^63^ Comparable accuracy has been found between perfusion amyloid‐PET and FDG‐PET in classifying participants into AD or other neurodegenerative diseases.^58^, ^59^, ^61^ Moreover, correlations between perfusion amyloid and MMSE were similarly strong as those between FDG‐PET and MMSE.^64^

Similar to early‐phase amyloid‐PET, early‐phase tau‐PET allows for the assessment of the topographical distribution of both perfusion and tau pathology. Strong spatial association between early‐phase tau‐PET hypoperfusion assessed by [^18^F]Flortaucipir^65^ or [^18^F]PI‐2620^66^, ^67^ and reduced glucose metabolism measured with FDG‐PET has been widely demonstrated.^65^ Tau‐PET perfusion measures were also significantly correlated with MMSE scores in AD‐related regions. When visually assessed, there was a strong agreement between areas of reduced perfusion in early‐phase tau‐PET and hypometabolism in FDG‐PET, irrespective of the tau tracer.^65^, ^66^ Moreover, a robust agreement was found between the early phase of tau‐PET ([^18^F]PI‐2620) and of amyloid‐PET ([^18^F]Flutemetamol).^67^ Importantly, dual‐phase tau‐PET holds added value given its strong correlation with cognitive decline, which highlights the clinical relevance of the dual‐phase imaging.

Furthermore, the European Association of Nuclear Medicine highlights the dual‐phase acquisition of amyloid‐PET, which provides an additional measure of N in the AT(N) system, potentially useful also for differential diagnosis in clinical practice. Early phase acquisition may help to disentangle complex cases that have discrepant biological and clinical phases due to co‐pathologies. Moreover, early‐phase amyloid‐PET was able to detect different neurodegenerative patterns such as frontotemporal dementia and dementia with Lewy bodies.^61^, ^68^

The dual‐phase approach offers major clinical advantages. It allows for simultaneous assessment of both protein deposition and cerebral perfusion from a single scan; it reduces radiation exposure, shortens diagnostic time and potentially enables earlier diagnosis and treatment monitoring.^57^ Overall, this supports the integration of early‐phase PET into clinical workflows as a practical and efficient alternative to separate FDG‐PET scans.

#### 3.1.3 Synaptic density

Synapses are critical structures that allow communication between neurons and are essential for maintaining cognitive function. Synaptic loss is a consistent pathological feature in AD and is strongly associated with cognitive impairment and memory deficits.^69^ Synaptic dysfunction emerges early in AD as one of the key pathological hallmarks, even before significant neuronal loss occurs. Synaptic vesicle glycoprotein 2A (SV2A), a glycoprotein located in the presynaptic vesicles of glutamatergic and GABAergic neurons throughout the central nervous system, serves as a valuable biomarker for synaptic density.^70^, ^71^ Imaging SV2A using PET tracers such as [^11^C]UCB‐J, [^11^C]UCB‐H and [^18^F]SynVesT‐1 revealed marked reductions in synaptic density in AD‐vulnerable regions, including the hippocampus, posterior cingulate and temporal cortices, even during the prodromal stages of the disease.^70^, ^71^, ^72^ These alterations become more widespread, extending to frontal and parietal regions as the disease advances.

Amyloid‐related synaptic loss was supported through [^18^F]UCB‐H PET imaging, which highlighted substantial hippocampal reductions in synaptic density among amyloid‐positive individuals.^72^ Similarly, reduced SV2A binding was observed in regions such as the entorhinal cortex, implicating early degeneration.^69^ Furthermore, SV2A PET measures were linked to amyloid and tau pathologies, glucose hypometabolism and cognitive decline across diverse populations, including young adults, cognitively normal individuals and cognitively impaired AD patients.^73^, ^74^ Higher tau burden, measured via [^18^F]Flortaucipir PET, is linked to decreased [^11^C]UCB‐J binding in CSF amyloid‐positive individuals.^75^ Likewise, an inverse relationship between global amyloid deposition assessed with PiB PET and synaptic density was observed using [^11^C]UCB‐J, particularly pronounced during the MCI stage when more amyloid accumulation is present; moreover, amyloid and tau were inversely associated with hippocampal synaptic density in MCI and mild AD patients.^76^ A comprehensive study used [^11^C]UCB‐J PET in combination with neuropsychological batteries also revealed that lower global synaptic density was correlated with poorer performance across cognitive domains in early AD, suggesting that synaptic PET may serve as a sensitive indicator of cognitive dysfunction.^77^ These findings were consistent with studies using [^18^F]SynVesT‐1, which reported reduced synaptic density in both cortical and hippocampal areas in individuals with MCI and AD dementia compared to controls, with significant changes in the right insular cortex and bilateral caudal middle frontal gyrus, regions where synaptic loss was directly associated with cognitive decline.^78^ Combined assessments using [^11^C] UCB‐J and [^18^F] FDG revealed stronger inter‐tracer correlations between synaptic density and glucose metabolism in the medial temporal regions, whereas weaker correlations were observed in neocortical areas in AD patients. This can suggest region‐specific coupling between synaptic density and glucose metabolism.^74^

Collectively, these converging findings suggest the utility of SV2A PET as a powerful approach for capturing synaptic alterations in relation to amyloid and tau pathology, metabolic changes and cognitive performance. It offers a more comprehensive understanding of the early and progressive synaptic disturbances in AD. This tool would be particularly useful to monitor treatments' impact on synaptic activity and neuroprotective approaches, important targets in the current AD treatment landscape.^79^

Furthermore, loss of synaptic density may not be specific only to AD‐related neurodegeneration^80^, ^81^, ^82^; thus, the regional pattern of synaptic loss could potentially provide insights for differentiating various types of dementia. SV2A PET imaging has promising clinical applications, including early detection of synaptic dysfunction, differential diagnosis across dementia types and longitudinal monitoring of disease progression.^83^

### 3.2 Neuroinflammation

#### 3.2.1 Microglia

Microglial activation is one of the earliest events in AD and plays a complex and important role in disease progression, as microglia have different subtypes of active microglia which depend on the gene expression.^84^ Microglia can be expressed and can exert neuroprotective (M2 profile) and neurotoxic effects (M1 profile).^84^, ^85^ In early stages, microglia promote amyloid clearance through phagocytosis, helping to limit oligomer formation and amyloid‐related toxicity.^18^ However, when clearance fails, microglial activation shifts to a proinflammatory state, releasing neurotoxic cytokines that drive neurodegeneration.^86^, ^87^ This inflammatory transition is driven by altered immune cytokine activities that intensify during AD progression. The main molecular imaging target for activated microglia is the translocator protein (TSPO), primarily expressed on the outer mitochondrial membrane of steroid‐synthesizing cells in the central nervous system (CNS).^77^ PET with first‐generation tracers for TSPO such as [^11^C]PK11195 offered important initial insights, but studies were hampered by major tracer limitations, such as low blood–brain barrier permeability and high non‐specific plasma binding, resulting in a poor signal‐to‐noise ratio. Second‐generation tracers such as [^11^C]PBR28, [^11^C]DPA‐713, [^18^F]FEDAA1106 and [^18^F]FEPPA demonstrated improved specificity, higher brain penetration and better pharmacokinetics, enhancing the detection of subtle changes in TSPO expression.^88^, ^89^

Multimodal PET studies investigated associations between TSPO binding and other biomarkers, such as amyloid accumulation, tau burden and neurodegeneration. Topographic overlap and significant correlation were observed between elevated [^11^C]PK11195 binding and reduced brain metabolism measured with FDG‐PET in patients with MCI. These findings suggest that microglial activation was present in the prodromal stage of the disease and may contribute to early neurodegeneration.^90^, ^91^ Microglial activation was strongly correlated with tau aggregation, and the correlation was stronger in AD dementia compared to MCI, especially in regions of advanced Braak stages.^92^ Another study found a lack of correlation between tau accumulation and microglia in early AD, suggesting neuroinflammation as an earlier phenomenon linked to amyloid pathology at the beginning of the disease.^93^, ^94^ Accordingly, Pascoal et al.^95^ demonstrated that amyloid pathology potentiated microglial activation measured by [^11^C]PBR28 which in turn promotes tau propagation across the cortex.

On the contrary, some studies observed that microglial activation was elevated in early AD but tends to decrease as individuals progress from MCI to dementia,^96^ supporting microglia's protective role in the beginning of the continuum. Higher TSPO binding measured with [^18^F]DPA‐714 was associated with better cognitive performance, greater grey volume, slower clinical decline over 2 years, particularly in prodromal AD patients.^97^ Similar results were observed with [^11^C]PK11195 which supports a bimodal pattern of microglial activation in AD: microglial activity may reflect a protective response in prodromal stages, whereas it shifts to a proinflammatory phenotype in later stages.^90^, ^98^ Together, these results emphasized the complex and dynamic role of microglial activation in AD. The main limitation is the uncertainty around whether microglial activation plays a protective or worsening role in the pathology by having different subtypes, which current microglia biomarkers are unable to distinguish.^84^

#### 3.2.2 Astrocytes

Amyloid deposits in AD can trigger the activation of astrocytes, leading to an overexpression of cytokines and other cytotoxic molecules, ultimately resulting in an inflammatory response and oxidative stress. Astrocytes normally support endothelial cells of the blood–brain barrier, maintain ion balance and provide nutrients to surrounding neurons, undergoing significant changes during these processes. In AD, an increased number of astrocytes, known as astrogliosis, is observed around amyloid deposits. In pathological conditions, astrocytes undergo both morphological and functional changes and become what are referred to as reactive astrocytes.^99^ This reactive state of astrocytes can cause damage and dysfunction in their interactions with neighbouring neurons, disrupt synaptic homeostasis and initiate a cascade of neuronal injury.^85^ The activation of reactive astrocytes can be measured with the overexpression of glial fibrillary acidic protein (GFAP) and by imaging techniques targeting monoamine oxidase‐B (MAO‐B).

The PET radiotracer [^11^C]DED is considered the gold standard for in vivo imaging of reactive astrogliosis through MAO‐B binding.^100^ However, [^11^C]DED has limitations including irreversible kinetics, radiolabelled metabolites that can cross the blood–brain barrier, poor image quality, low selectivity for MAO‐B.^101^ Additionally, newer tracers such as [^18^F]SMBT‐1 offer high blood–brain barrier permeability, reversible kinetics and low non‐specific binding.^101^ [^18^F]SMBT‐1 is the first F‐18 MAO‐B radiotracer available to be used in clinical research settings to assess reactive astrogliosis.

Evidence from multiple PET studies targeting astrocytosis suggests that reactive astrocyte activity emerges early in the disease and appears closely linked to amyloid pathology.^102^, ^103^, ^104^ Increased [^18^F]SMBT‐1 uptake was observed in regions typically affected in early AD, such as the posterior cingulate, supramarginal gyrus, frontal and parietal cortices. These regional patterns of binding are often more pronounced in individuals with amyloid positivity, also when assessed with [^11^C]DED,^103^, ^104^ suggesting that astrocyte reactivity may be a response to early amyloid accumulation. This is further supported by the evidence that uptake is highest in amyloid positive MCI patients compared to CU and dementia subjects, which demonstrates astrogliosis as a potential early and transient event in disease progression.^102^, ^103^, ^104^

However, the precise role and timing of neuroinflammation remain complex and dynamic in the disease continuum. Previous evidence suggests presence of two peaks of astrocyte reactivity, with an early peak during presymptomatic or prodromal stages that may affect amyloid positivity, followed by a second peak in advanced AD dementia that contributes to neurotoxic effects, tau spreading and cognitive decline.^105^ In studies with autosomal dominant AD (ADAD) caused by mutations in the Presenilin 1, Presenilin 2 and amyloid precursor protein genes, astrocyte activation has been detected up to 17 years before symptom onset.^106^ Highest [^11^C]DED binding was present in presymptomatic mutation carriers and coincided with initial stages of amyloid deposition.^104^, ^106^, ^107^

Several preclinical studies support the relevance of astrocytes and microglia in the disease onset and progression, but evidence in clinical studies is still limited. Although TSPO PET remains the most widely used neuroinflammation technique in patient cohorts, it is limited by its lack of cellular specificity and inability to discriminate between beneficial and toxic immune responses, which must be considered when interpreting the results.^108^ Although neuroinflammation is recognized as a critical component of AD pathophysiology, particularly in light of emerging therapeutic strategies, the implementation of specific PET biomarkers into clinical practice remains challenging. A major limitation is the lack of specificity of neuroinflammatory biomarkers, which can be elevated in a variety of neurological and systemic conditions beyond AD.^109^ This limited specificity may arise from astrocyte heterogeneity in AD. Astrocyte reactivity appears to involve multiple phenotypes and subtypes that may vary across disease stages.^110^ Although further efforts are needed to translate them into clinical practice, neuroinflammation tracers show strong correlations with cognitive decline and could serve as valuable tools for monitoring therapeutic responses.

## 4 BIOMARKERS FOR CO‐PATHOLOGY

### 4.1 Alpha‐synuclein

Alpha‐synuclein plays a crucial role in synaptic vesicle dynamics and neurotransmitter release, and its pathological aggregation is often observed as a co‐pathology in AD. It exists in both soluble and membrane form and is expressed in brain regions such as the striatum, cortex, hippocampus and thalamus.^111^ In pathological conditions, alpha synuclein becomes abnormally phosphorylated and aggregates into neurotoxic forms such as fibrils, which form Lewy bodies. The pathology is the occurrence of intracellular Lewy bodies and Lewy nutrients containing alpha synuclein aggregates.^112^ Alpha‐synuclein may interact with amyloid and tau, and leads to the exacerbation of AD. AD often occurs together with dementia with Lewy bodies (DLB); comorbidity has been reported in more than half of postmortem evaluations of AD.^112^ Moreover, AD subjects with evidence of alpha synuclein deposits had an accelerated cognitive decline compared to pure AD.^111^

The development of a radioligand for alpha‐synuclein imaging has been very challenging due to the relatively low density of its fibrillar aggregates in brain tissue, which falls below the current spatial resolution limits of PET imaging.^113^ Compared to amyloid and tau, alpha‐synuclein is also present in much lower concentrations and thus requires the development of tracers with high binding affinity and selectivity. However, achieving binding specificity is complicated due to the structural similarity of alpha‐synuclein aggregates to those of amyloid and tau, as they all share a beta‐sheet rich conformation. Among investigated tracers, [^18^F]F0502B showed the highest specificity for alpha‐synuclein in human brain slices.^114^ Another promising tracer is [^18^F]C05‐05, which demonstrated high affinity and sustained retention of free and bound compounds in the brain, with greater binding affinity to alpha‐synuclein over other proteins.^115^

Imaging in patients with Parkinson's disease dementia (PDD), DLB and controls showed increased [^18^F]C05‐05 retention in the midbrain of DLB patients, in the putamen and middle cerebellar peduncles of subjects with multiple system atrophy (MSA), which correlates with motor symptoms.^116^, ^117^ Specific binding of [^18^F]C05‐05 was observed in regions known for alpha‐synuclein deposition, such as the amygdala and substantia nigra, although off‐target binding to white matter and choroid plexus remains a concern. Another tracer, [^18^F]ACI‐12589, exhibited retention in the cerebellar white matter and cerebellar peduncles, was able to distinguish MSA patients from controls and PDD and DLB patients, with a significant discriminatory power for MSA diagnosis. This tracer shows no off‐target binding to other protein aggregates, which is a major asset for alpha‐synuclein imaging. Lack of retention in PD and DLB cases might be due to lower density of alpha‐synuclein pathology in these disorders compared to MSA.^118^ Current research suggests that alpha‐synuclein PET is not specific for diagnosis of Lewy body dementia (LBD); however, it shows potential in distinguishing MSA from other neurodegenerative diseases.^119^

Clinical studies have so far investigated only non‐AD populations by using PET imaging.^120^, ^121^ Most tracers are still in the early validation and preclinical stage; therefore, further development and refinement of these tracers are essential to enable their clinical validation and ultimately use in detecting alpha‐synuclein pathology. The development of reliable tracers for alpha‐synuclein could support pathophysiological investigation as well as the diagnosis and management of individuals with synucleinopathies.

### 4.2 TDP43

TAR DNA‐binding protein 43 (TDP‐43) aggregation is a hallmark of several neurodegenerative diseases, including amyotrophic lateral sclerosis (ALS), PD, frontotemporal dementia (FTD), AD and limbic predominant age‐related TDP‐43 encephalopathy (LATE). In healthy cells, TDP‐43 is predominantly located in the nucleus, where it plays a crucial role in RNA regulation; however, under pathological conditions, it undergoes cleavage, hyperphosphorylation and ubiquitination, leading to its mis‐localization into the cytoplasm, accumulation and aggregation.^122^, ^123^

TDP‐43 depletion has been shown to amplify microglial activation and contribute to synaptic loss and neurodegeneration.^124^ In AD, TDP‐43 often appears as a secondary co‐pathology, and its aggregation is linked to soluble amyloid oligomers that may promote TDP‐43 aggregation through cross‐seeding mechanisms.^125^ In the absence of in vivo specific markers for TDP‐43, molecular imaging studies in LATE have so far used FDG‐PET imaging to detect hypometabolic patterns suggestive of the disease. TDP‐43 co‐pathology with and without AD co‐pathology has been associated with specific patterns of brain hypometabolism, particularly in the medial temporal and frontal regions.^125^ In particular, the ratio between inferior and medial temporal regions' metabolism has been used as a summary marker for LATE, achieving a sensitivity of 81% and specificity of 74% in distinguishing TDP‐43 positive cases with postmortem confirmation from AD.^126^, ^127^ Nevertheless, the development of a specific radiotracer for TDP‐43 is crucial for accurate diagnosis, better patient categorization and the advancement of effective therapeutic strategies. Difficulties in creating a radiotracer for TDP‐43 are mainly due to its intracellular location and limited neuropathologic load.^113^ Recently, the first two TDP‐43‐selective tracers, [^18^F]ACI‐19626 and [^18^F]ACI‐19278, demonstrated high‐affinity binding to aggregated TDP‐43 derived from FTLD‐TDP brain samples, with good selectivity over amyloid‐beta, tau and alpha‐synuclein aggregates, and no off‐target binding to monoamine oxidase.^128^ Given their good brain uptake and fast washout properties, [^18^F]ACI‐19626 and [^18^F]ACI‐19278 look like promising PET imaging agents for detecting and monitoring TDP‐43 pathology in vivo in proteinopathies such as FTLD‐TDP, AD and LATE.^129^, ^130^

However, these findings are preliminary and primarily derived from preclinical or postmortem studies. Clinical studies with tracers targeting TDP‐43 using an effective tracer are still lacking and would be of great clinical interest for the differential diagnosis of TDP‐43 proteinopathies.^123^

## 5 MOLECULAR IMAGING IN TREATMENT SELECTION, MONITORING AND FUTURE PERSPECTIVES

Molecular imaging markers are now routinely incorporated into clinical trials, and the emergence of current and future disease‐modifying therapies is expected to transform the role and implications of diagnostic procedures in AD.

Three anti‐amyloid immunotherapies: aducanumab,^131^ lecanemab^132^ and donanemab,^133^ have used amyloid‐PET as a baseline inclusion biomarker and a surrogate endpoint to monitor plaque clearance. These studies have shown that reductions in amyloid burden on PET are associated with modest but significant slowing of cognitive decline. Both lecanemab and donanemab received FDA approval. They represent significant progress in anti‐amyloid immunotherapies despite ongoing discussions on safety, clinical significance and patient selection. In current therapies and clinical trials for AD, tau‐PET might have a critical role for screening participants that would benefit more from anti‐amyloid targeting therapies, as demonstrated for donanemab.^133^ There are still some challenges when including therapies in clinical practice. One major obstacle is the need for early but also accurate diagnosis for individuals to benefit from these trials. The combination of plasma biomarkers as a screening tool to identify subjects at risk and multitracer PET‐based assessment could further improve our ability to correctly characterize patients' heterogeneity.^134^

## 6 CONCLUSION

This review summarized the wide range of PET biomarkers available in research and clinical settings targeting both specific and non‐specific pathological processes in AD. Amyloid and tau‐PET imaging have advanced the field by enabling in vivo visualization of pathologies, supporting early and differential diagnosis, as well as prognosis, and offering objective endpoints for clinical trials. Complementary non‐specific biomarkers, such as FDG‐PET for neurodegeneration and emerging tracers that target synaptic function or neuroinflammation, provide further insight into the processes occurring in disease progression and contribute to explaining the heterogeneity of the disease. Additionally, molecular imaging targeting co‐pathologies, including α‐synuclein and TDP‐43, may enhance diagnostic precision in atypical or mixed clinical presentations. PET imaging has been instrumental in investigating and evaluating the complete disease process of AD. Longitudinal PET scans can assess therapies' effectiveness and mechanisms of action over time. As the field continues to grow, research priorities should be on the direction of integrating multiple PET biomarkers within a personalized, biologically driven diagnostic framework. Future research should aim to validate novel tracers and diagnostic algorithms, improve standardization across imaging and explore longitudinal changes in biomarkers.

## AUTHOR CONTRIBUTIONS

EH, GM, MJ, NT were responsible for drafting the original report, which was reviewed by CB, JOP and VG.

## FUNDING INFORMATION

VG received funding for this research from the Swiss National Science Foundation (projects 320030_169876, 320030_185028 and 320030_220099), Velux Fonden, Schmidheiny Foundation and Fondation Privée des HUG.

## CONFLICT OF INTEREST STATEMENT

VG received research support and speaker fees through her institution from GE Healthcare, Siemens Healthineers, Novo Nordisk, Janssen and Novartis. GBF has received support, payment, consulting fees or honoraria through his institution for lectures, presentations, speaker bureaus, manuscript writing or education events from Biogen, Roche, Diadem, Novo Nordisk, GE Healthcare, OM Pharma and Eisai. EH, CB, GM, MJ, NT, JOP report no conflict of interests. There are no conflicts of interest related to the study design or the results.

## Data Availability

Data sharing not applicable to this article as no datasets were generated or analysed during the current study.
